# Louisiana Geriatric Workforce Enhancement Program: Analysis of Learning Outcomes From Dementia Training

**DOI:** 10.1093/geroni/igaa057.189

**Published:** 2020-12-16

**Authors:** Katherine Kirsch, Catherine Lemieux, Laura Ainsworth, Sarah Choate, Ashleigh Borgmeyer, Cassie Slaton, Scott Wilks

**Affiliations:** 1 Louisiana State University, Baton Rouge, Louisiana, United States; 2 Louisiana State University, Hattiesburg, Mississippi, United States; 3 Louisiana State University, Ocean Springs, Mississippi, United States

## Abstract

A recent Alzheimer’s Association report noted that by year 2050, the number of Americans diagnosed with Alzheimer’s disease and related dementias (ADRD) will triple to over 15 million. The report referred to primary care as the front line for meeting this demand, yet the nation faces a severe shortage of ADRD trained, primary care professionals (PCPs). Louisiana Geriatric Workforce Enhancement Program (LA-GWEP) addresses this demand. The purpose of this study was to examine preliminary data respective to LA-GWEP effectiveness with interdisciplinary education and training seminars, primarily aimed at medical, nursing, and social work PCPs. Three seminars were conducted in south Louisiana: Seminars 1 and 2 addressed effective communication, verbal and nonverbal, among persons with ADRD and caregivers; Seminar 3 offered basic overview of dementia symptomology, stages, and behaviors. Pre- and post-training session data were collected on-site. Participants completed questionnaires that included a 10-item knowledge assessment and 20-item Dementia Attitudes Scale (DAS). These measures contained Likert response formats; higher scores indicating greater levels of ADRD knowledge, in patient and caregiver contexts. Paired sample t-tests were conducted to observe any significant pre-to-post improvement, Cohen’s d for effect size. Seminar 1 revealed no significant pre-to-post difference: t = -1.019, p = 0.320. Adjusting content from audience feedback, Seminar 2 revealed significant pre-to-post difference: t = -7.516, p < .001, Cohen’s d = 1.2. Seminar 3 yielded significant improvement on DAS scores: t = -2.96, p < .01, Cohen’s d = 0.34. Implications for seminars in future years of LA-GWEP are discussed.

